# Supplementary material for: Temporal ablation of the ciliary protein IFT88 alters normal brainwave patterns
Source: Sci Rep. 2025 Jan 2;15:347. doi: 10.1038/s41598-024-83432-1 (PMC11697071; doi:10.1038/s41598-024-83432-1)
Supplement: Supplementary file 1 — Supplementary Material 1 [file 41598_2024_83432_MOESM1_ESM.docx]

**Supplementary figure**

**Figure S1:** **Manual scoring of mouse EEG/EMG electrophysiological recordings.** (**a.**) Example of how 24-hour recordings were cluster-scored in Sirenia Sleep 1.0.3 by comparing EEG to EMG signal strength to identify clusters of Wake, Non-REM, and REM data. (**b.**) The recordings were manually curated in 10 second epochs to correct cluster scoring. The awake state is characterized by activity in the occipital lobe (EEG1), high frequency-low amplitude activity in the frontal lobe (EEG2), and high levels of EMG activity. Asleep mice lack EMG and occipital lobe activity. The non-REM state is identified by low frequency/high amplitude brain waveforms and REM sleep is differentiated by high frequency-low amplitude EEG activity, similar to what is seen while awake.
